# Comparative Study on Pervaporation Performance of Polyphosphazene Membranes with Different Fluorine Side Groups for Thiophene/*n*-Heptane Separation

**DOI:** 10.3390/polym17111573

**Published:** 2025-06-05

**Authors:** Bingcong Xu, Xingmei Zhang, Wenwen He, Xiaolong Han

**Affiliations:** 1 School of Chemical Engineering, Northwest University, Xi’an 710069, China; xbc13483335337@163.com; 2 School of Chemical Engineering, Xi’an University, Xi’an 710065, China; 3 School of Chemistry and Life Science, Advanced Institute of Materials Science, Changchun University of Technology, Changchun 130012, China

**Keywords:** pervaporation desulfurization, polyphosphazene, composite membranes

## Abstract

In recent years, polyphosphazene (POP) membranes have been gaining more and more attention owing to their excellent pervaporation desulfurization performance. To develop new POP membranes, three kinds of POPs with different side groups, Poly[bis(trifluoroethoxy)phosphazene] (PTFEP), Poly[bis(trifluorobutoxy)phosphazene] (PTFBP), and Poly[bis(octafluoropentyloxy)phosphazene] (POFPP), were synthesized. The NMR spectroscopy demonstrated that POPs with a designed structure were successfully prepared. Subsequently, the composite membranes based on these POPs were fabricated by solution casting. The influence of side groups on the desulfurization performance of membranes was systematically evaluated via a pervaporation test. Among these membranes, the PTFBP membrane exhibited the highest separation efficiency, significantly outperforming other membrane types with a permeation flux of 0.284 kg·m^−2^·h^−1^ at 200 ppm and 85 °C, along with a sulfur enrichment factor of 26.48. In addition, the effects of temperature and feed concentration on separation performance were investigated in detail.

## 1. Introduction

In recent years, as society has developed, gasoline has become a crucial energy source, providing significant convenience for daily life. However, the adverse environmental impacts resulting from gasoline combustion have emerged as a pressing global concern. The sulfur oxides (SO_x_) generated from the combustion of sulfur in gasoline contribute to the formation of acid rain when released into the atmosphere. Furthermore, the utilization of gasoline containing high sulfur levels can diminish the efficiency of catalysts in vehicle exhaust purification systems [1]. Addressing the sulfur content in gasoline has become an urgent priority. The newly enforced National VIB standard for “*Gasoline for motor vehicles*” [2] in China stipulates that the sulfur content in gasoline should not exceed 10 mg/kg. Notably, fluid catalytic cracking (FCC) gasoline constitutes 70% of China’s automotive gasoline pool [3]. Within FCC gasoline, 20% of the sulfur is present in a removable form, such as mercaptan sulfur, while the remaining 80% exists as challenging-to-remove sulfur compounds, like thiophene sulfur [4,5]. Consequently, the primary focus of gasoline desulfurization efforts lies in eliminating thiophene sulfur from gasoline.

Various methods for gasoline desulfurization are available, including adsorption desulfurization [6], extraction desulfurization [7], biological desulfurization [8], oxidative desulfurization [9], alkylation desulfurization [10], hydrodesulfurization [11], and pervaporation (PV) desulfurization. The conventional hydrodesulfurization and adsorption desulfurization methods encounter challenges, such as high energy consumption, significant octane loss, frequent adsorbent replacement, and so on [12]. In contrast, pervaporation desulfurization has emerged as a preferred technique due to its straightforward operation, low energy requirements, and negligible octane loss [13]. The effectiveness of pervaporation desulfurization fundamentally relies on the pervaporation separation membrane, which is pivotal in determining the separation efficiency. Current pervaporation desulfurization membranes are primarily composed of polyimide (PI) [14], polyethylene glycol (PEG) [15], poly(ether-block-amide) (PEBAX) [16], polydimethylsiloxane (PDMS) [17], and ethylcellulose (EC) [18]. However, these membranes often exhibit issues such as inadequate mechanical stability, restricted chemical modification options, and low separation performance [19,20]. Consequently, the quest for a highly efficient membrane material has emerged as a primary focus in pervaporation desulfurization research.

Polyphosphazene (POP) features a main chain structure with alternating phosphorus-nitrogen single and double bonds. The successful synthesis of the linear polymer Poly(dichlorophosphazene) (PDCP) by Allcock [21] marked a significant milestone. Notably, the chlorine atoms present on the side groups of PDCP exhibit high reactivity, enabling easy substitution by nucleophilic reagents to achieve modified POP. This transformation has significantly enhanced the structural diversity of POP [22,23,24,25,26] and facilitated the manipulation of its properties by altering the side group composition. According to the solubility parameter theory [27,28], Yang et al. [29] selected PTFEP as a desulfurization membrane and found that the sulfur enrichment factor of the PTFEP membrane surpassed that of other membrane materials because the solubility parameter of PTFEP closely aligns with that of thiophene. Based on the structural diversity of POP, we think that incorporating other fluorine-containing side groups into POP may yield unforeseen outcomes.

Therefore, three types of POPs featuring various fluorine-containing side groups, Poly[bis(trifluoroethoxy)phosphazene] (PTFEP), Poly[bis(trifluorobutoxy)phosphazene] (PTFBP), and Poly[bis(octafluoropentyloxy)phosphazene] (POFPP), were synthesized. Subsequently, the corresponding composite membranes of PTFEP/PVDF, PTFBP/PVDF, and POFPP/PVDF were fabricated on a PVDF support layer using the coating method. Furthermore, the pervaporation desulfurization performance of various composite membranes was comprehensively evaluated, and the effects of fluorine content and fluorine distribution within POP side groups on membrane desulfurization performance were investigated.

## 2. Materials and Methods

### 2.1. Fabrication of Membranes

#### 2.1.1. Synthesis of POP Materials

**Figure 1:**
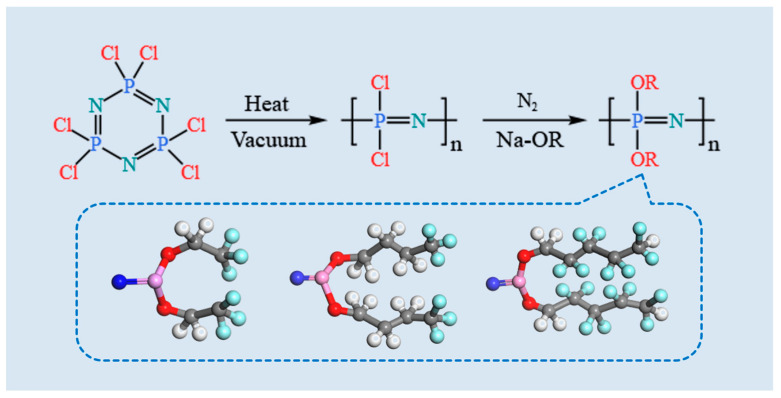
Synthesis process of three kinds of POPs.

The synthesis of the POP was carried out according to the procedure described in the reference [29]. Pure Hexachlorocyclotriphosphazene (HCCP) was ring-opened polymerized to produce the intermediate PDCP. Subsequently, pre-prepared sodium trifluorobutyl was added dropwise to the solution of PDCP in anhydrous tetrahydrofuran (THF), and the reaction was allowed to proceed for 48 h. Finally, pure PTFBP was obtained following purification steps. PTFEP and POFPP were synthesized using the same process. The detailed synthesis process is illustrated in Figure 1.

#### 2.1.2. Preparation of Homogeneous Membranes

The homogeneous membrane was prepared by dissolving synthesized POP in THF to achieve a 20 wt% solution. This solution was then poured onto a clean glass plate and dried at room temperature and 60 °C in an oven for 24 h to allow solvent evaporation. The resulting homogeneous POP membranes were then stored for further characterization.

#### 2.1.3. Preparation of Composite Membranes

**Figure 2:**
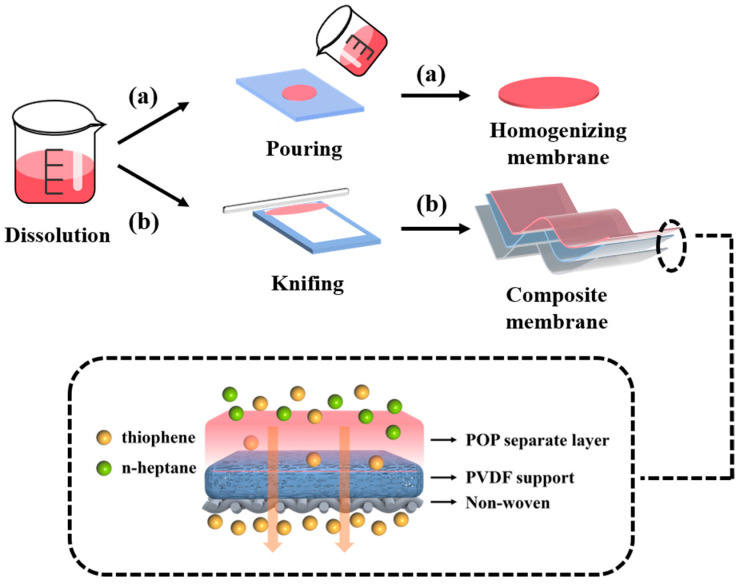
Schematic diagram of preparation process: (a) homogenizing membrane and (b) composite membrane.

The composite membrane was prepared by dissolving synthesized POP in THF to achieve a 10 wt% solution. Next, the PVDF membrane was cut to the appropriate size and affixed to a clean glass plate. The THF solution of POP was cast onto the PVDF membrane. Subsequently, the membrane was dried at room temperature for 72 h and at 60 °C for 24 h to ensure complete evaporation of the solvent. The resulting composite membranes were then stored under dry conditions for further characterization. The detailed preparation steps for these membranes are illustrated in Figure 2.

### 2.2. Characterization and Pervaporation Test

The elemental chemical environment of the polymers was analyzed using NMR (ECZ600R, JEOL, Tokyo, Japan). The thermal stability, glass transition temperature (T_g_), and swelling degree (SD) of the homogeneous membranes were determined through TGA (HJR-1) and DSC (DSC 3500, NETZSCH, Selb, Germany). The surface composition, surface morphology, and cross-section morphology of the composite membranes were examined using FT-IR (IRAffinity-1S, Shimadzu, Kyoto, Japan), XPS (ESCALAB Xi^+^, Thermo Scientific, Waltham, MA, USA), SEM (Apreo S, Thermo Scientific, Waltham, MA, USA), and contact angles (V1, Yunfan, Tianjin, China). The desulfurization performance of the composite membranes was assessed using a laboratory-made pervaporation device [30], where the pressure on the downstream side of the membrane was maintained at about 200 Pa or 800 Pa.

## 3. Results and Discussion

### 3.1. Characterization

#### 3.1.1. NMR

**Figure 3:**
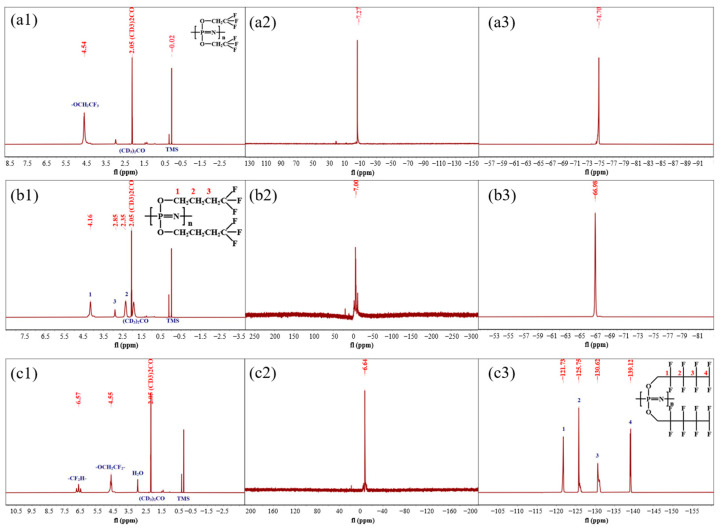
(**a1**,**c1**) 1H NMR; (**a2**,**c2**) 31P NMR; (**a3**,**c3**) 19F NMR spectra; (**a**) PTFEP/PVDF; (**b**) PTFBP/PVDF; (**c**) POFPP/PVDF.

The ^1^H NMR, ^31^P NMR, and ^19^F NMR spectra of PTFEP, PTFBP, and POFPP are shown in Figure 3. The letters (a), (b), and (c) in Figure 3 correspond to the ^1^H NMR, ^31^P NMR, and ^19^F NMR spectra of the prepared POP, respectively. The numbers (1), (2), and (3) represent the NMR spectra of PTFEP, PTFBP, and POFPP, respectively. As shown in Figure 3(a1,b1,c1), the H chemical environments in the POP are as follows: (a1): 4.54 ppm (-CH_2_-), (b1): 4.16ppm (C_1_), 2.35ppm (C_2_), 2.85ppm (C_3_), (c1): 6.57 ppm (-CF_2_H-) and 4.55 ppm (-CH_2_-). In Figure 3(a2,b2,c2), a single peak is observed at −7.27 ppm, −7.00ppm, and −6.64 ppm, respectively, indicating a single chemical environment for the P atoms in PTFEP, PTFBP, and POFPP. The peaks in Figure 3(a3,b3,c3) correspond to different chemical environments of F in the polymers, and are as follows: (a3): −74.70 ppm (-CF_3_); (b3): −66.98ppm (-CF_3_); (c3): −121.73 ppm (C_1_); −125.75 ppm (C_2_); −130.62 ppm (C_3_); −139.12 ppm (C_4_); these align with the characterization results cited in references [29,31,32,33]. The NMR spectra data confirm that the fully substituted PTFEP, PTFBP, and POFPP polymers have been successfully synthesized.

#### 3.1.2. TGA

**Figure 4:**
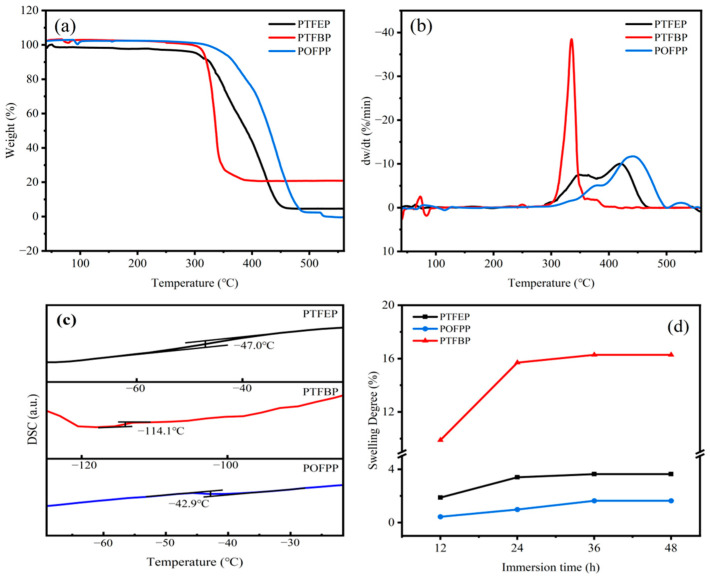
(**a**) TGA, (**b**) DTG, (**c**) DSC of POP, and (**d**) SD of POP membranes.

To ensure the stable separation performance of membranes during the pervaporation desulfurization process, the thermal stability of the polymer was assessed using TGA, which is shown in Figure 4a,b. The weight loss occurring before 303 °C can be attributed to the loss of small molecules and water, resulting in an average weight loss of 5%. The POP material initiates thermal decomposition within the temperature range of 303–348 °C, which is significantly higher than the temperature of the membrane during the pervaporation desulfurization process. Consequently, the composite membrane prepared by these POPs is capable of withstanding challenges such as decomposition and aging in practical utilization.

#### 3.1.3. DSC

The T_g_ of POP polymers was determined and is presented in Figure 4c. Specifically, the T_g_ values for PTFEP, PTFBP, and POFPP are −47.0 °C, −114.1 °C, and −42.9 °C, respectively. The T_g_ values follow the order of PTFBP < PTFEP < POFPP. It is important to note that the lower T_g_ values generally indicate enhanced mobility of molecular chain segments, leading to improved permeability of membranes.

#### 3.1.4. SD

The membrane tends to swell during use due to the favorable interaction between the polymer and thiophene. To assess the extent of swelling, the homogeneous membrane was immersed in a model gasoline containing 200 ppm of sulfur. The swelling degree (*SD*) was determined using Equation (1), as shown in Figure 4d. The SD values for the PTFEP, PTFBP, and POFPP homogeneous membranes all increased progressively with longer immersion times, ultimately stabilizing after 36 h at 3.63%, 16.28%, and 1.64%, respectively. The order of swelling degree among these membranes was POFPP < PTFEP < PTFBP. In general, the high swelling degree always means high permeability of the membrane.

(1) $$SD=\frac{M_{S}-M_{d}}{M_{d}}\times 100\%$$

where *M_S_* represents the mass of the homogeneous membrane after it was immersed in thiophene/*n*-heptane mixture for a certain period of time, and *M_d_* represents the initial mass of the homogeneous membrane.

#### 3.1.5. Homogeneous Membrane Morphology

**Figure 5:**
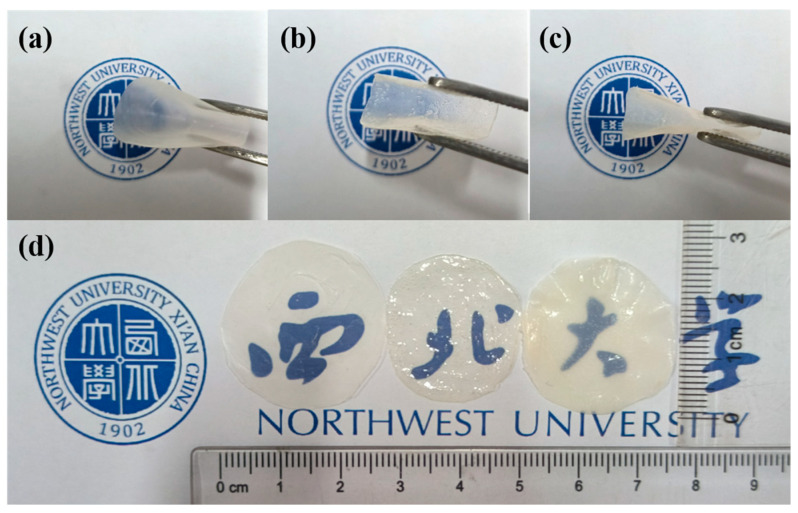
Images of the homogeneous membranes. (**a**) PTFEP, (**b**) PTFBP, (**c**) POFPP, and (**d**) the corresponding comparison of three POP membranes.

The morphology photographs of homogeneous membranes were taken using a camera, as depicted in Figure 5. Figure 5a–c displays the morphologies of homogeneous membranes made of PTFEP, PTFBP, and POFPP, respectively. It is evident that the homogeneous membranes are colorless and transparent with excellent flexibility. The diameter of these homogeneous membranes falls within the range of 2.5 cm to 3 cm, as illustrated in Figure 5d. From the morphology image of the homogeneous membranes, it is discernible that PTFEP, PTFBP, and POFPP all exhibit commendable film-forming properties.

#### 3.1.6. FT-IR

**Figure 6:**
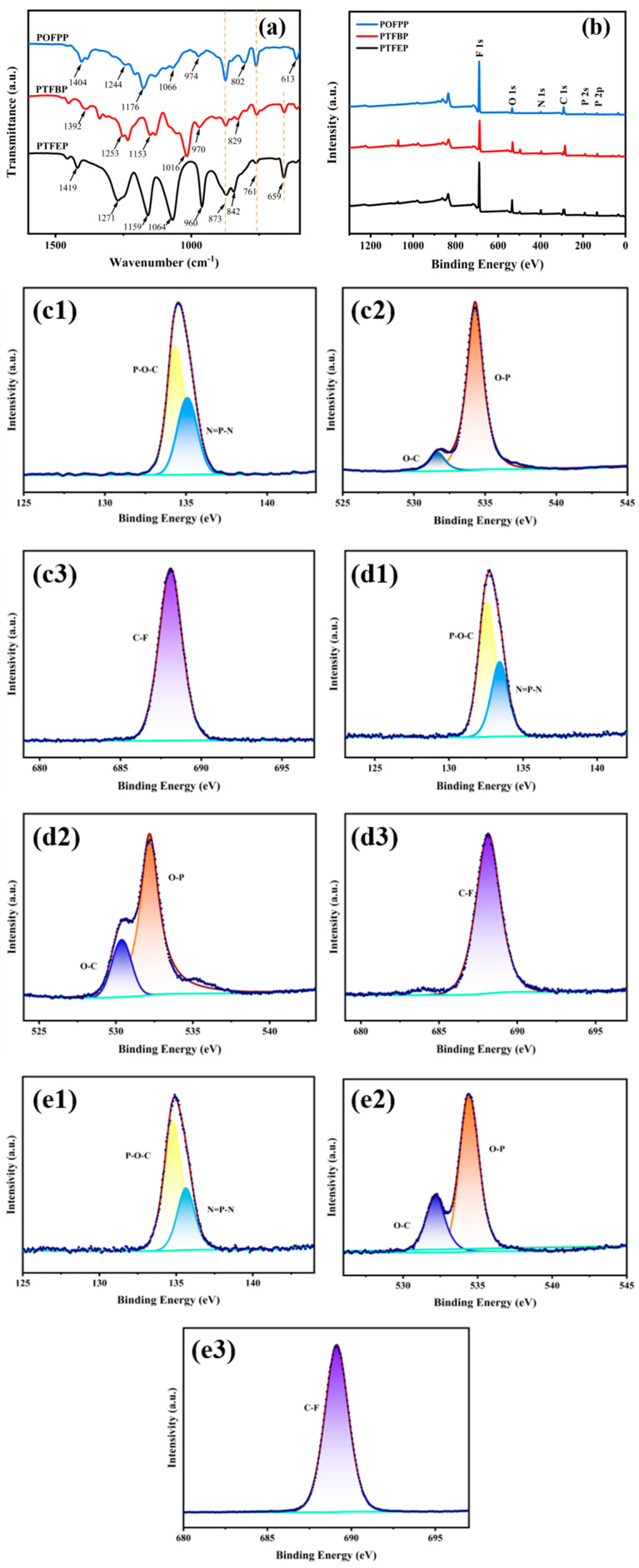
(**a**) FT-IR; (**b**) XPS; (**c**–**e**) High-resolution of XPS spectra; (**c1**–**e1**) P; (**c2**–**e2**) O; (**c3**–**e3**) F; (**c**) PTFEP/PVDF, (**d**) PTFBP/PVDF, and (**e**) POFPP/PVDF.

The FT-IR spectra of the composite membranes of PTFEP, PTFBP, and POFPP are presented in Figure 6a. Each group of PTFEP is associated with specific peaks: 1419 cm^−1^ (C-H), 1271 cm^−1^ (P=N), 1159 cm^−1^ (C-F), 1064 cm^−1^ (P-O-C), 960 cm^−1^ (P-O-C), 873 cm^−1^ (P-O-C), 842 cm^−1^ (P-N), 761 cm^−1^ (C-F), and 659 cm^−1^ (C-F). Notably, no characteristic P-Cl peaks at 579 cm^−1^ −590 cm^−1^ were observed, which is consistent with reference [31]. The peak positions in the FT-IR spectrum of PTFBP and POFPP closely aligned with those of PTFEP, and the peak deviations were within an acceptable range. This suggests that the composite membranes of PTFEP/PVDF, PTFBP/PVDF, and POFPP/PVDF were successfully prepared.

#### 3.1.7. XPS

The XPS spectrum of PTFEP/PVDF, PTFBP/PVDF, and POFPP/PVDF composite membranes is presented in Figure 6b. Figure 6b displays the peaks of F1s, O1s, N1s, C1s, P2s, and P2p, while the peaks of Cl atoms (~200 eV) are notably absent. The atomic proportions of each element in POP are detailed in Table 1, aligning with the anticipated theoretical ratios. In order to conduct a detailed analysis of the chemical bond composition present on the surface of the composite membrane, the high-resolution XPS spectra of the membrane were further characterized. The high-resolution of P, O, and F XPS spectra in POP are depicted in Figure 6. In Figure 6(c1,d1,e1), two peaks are observed, indicating the presence of the P–O–C bond at the intersection of the main chain and the side chain, as well as the N=P-N bond within the main chain. Additionally, Figure 6(c2,d2,e2) shows peaks corresponding to the O–C bond and the O–P bond at the linkage between the main chain and the side chain. In Figure 6(c3,d3,e3), a peak corresponding to the C–F bond on the side chain is depicted, indicating the successful preparation of the composite membrane.

#### 3.1.8. SEM

**Figure 7:**
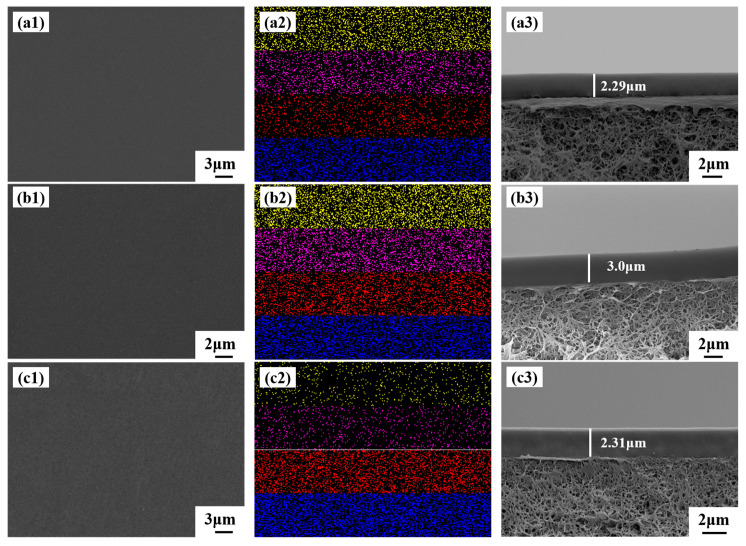
(**a1**–**c1**) Surface images, (**a2**–**c2**) mapping images, and (**a3**–**c3**) cross-sectional SEM images of membranes. P (yellow), O (pink), C(red), and F(blue). (**a**) PTFEP/PVDF, (**b**) PTFBP/PVDF, and (**c**) POFPP/PVDF.

The surface, surface mapping, and cross-section SEM images of the prepared PTFEP/PVDF, PTFBP/PVDF, and POFPP/PVDF composite membranes are displayed in Figure 7. Analysis of Figure 7(a1,b1,c1) reveals that the composite membranes exhibit a dense and smooth surface without discernible defects. In Figure 7(a2), Figure 7(b2), and Figure 7(c2), the yellow, pink, red, and blue colors correspond to the P, O, C, and F elements present on the membrane surface, respectively, demonstrating the uniform distribution of these elements across the membrane surface. Notably, the carbon content on the surface of the PTFEP, PTFBP, and POFPP composite membranes increases progressively, with the fluorine content being significantly higher in the POFPP composite membrane compared to the others. Figure 7(a3,b3,c3) shows that the separation layer thickness of the PTFEP, PTFBP, and POFPP composite membranes is approximately 2.2–4.2 μm. Additionally, the separation layer of these membranes exhibits a close combination with the support layer, a distinct interface, and an absence of apparent defects.

#### 3.1.9. Contact Angles

**Figure 8:**
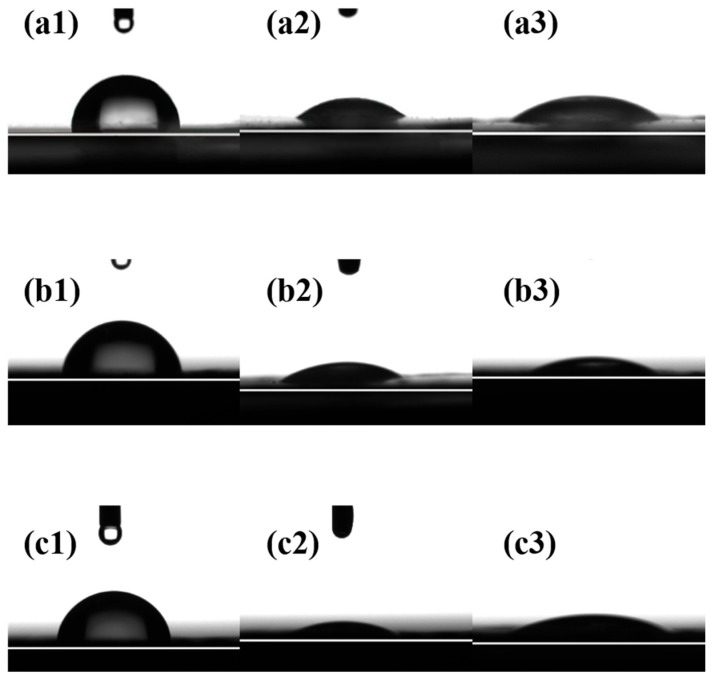
(**a1**–**c1**) Contact angle of water image, (**a2**–**c2**) contact angle of thiophene image, and (**a3**–**c3**) contact angle of n-heptane. (**a**) PTFEP, (**b**) PTFBP, and (**c**) POFPP.

The wettability of the composite membrane surfaces with different liquids was characterized by measuring the contact angles between the three composite membranes and water, thiophene, and *n*-heptane, as illustrated in Figure 8. The larger the contact angle between a droplet and the composite membrane surface, the poorer the wettability of the composite membrane toward the liquid. A contact angle of 90° serves as the dividing line: values greater than 90° indicate a lyophobic surface. As shown in Figure 8(a1,b1,c1), the water contact angles of all composite membranes are greater than 90°, indicating that the surfaces of the three composite membranes exhibit hydrophobic properties. This can be attributed to the distributed fluorine element on the PTPEP, POFPP, and PTFBP composite membrane surface. The wettability of the composite membrane surfaces with thiophene and *n*-heptane followed the order of PTFEP < POFPP < PTFBP, with thiophene exhibiting better wettability than *n*-heptane on the membrane surface.

### 3.2. Pervaporation Performance

#### 3.2.1. Influence of Feed Temperature

The pervaporation desulfurization performance of each composite membrane was evaluated under a constant feed concentration of thiophene at about 200 ppm.

**Figure 9:**
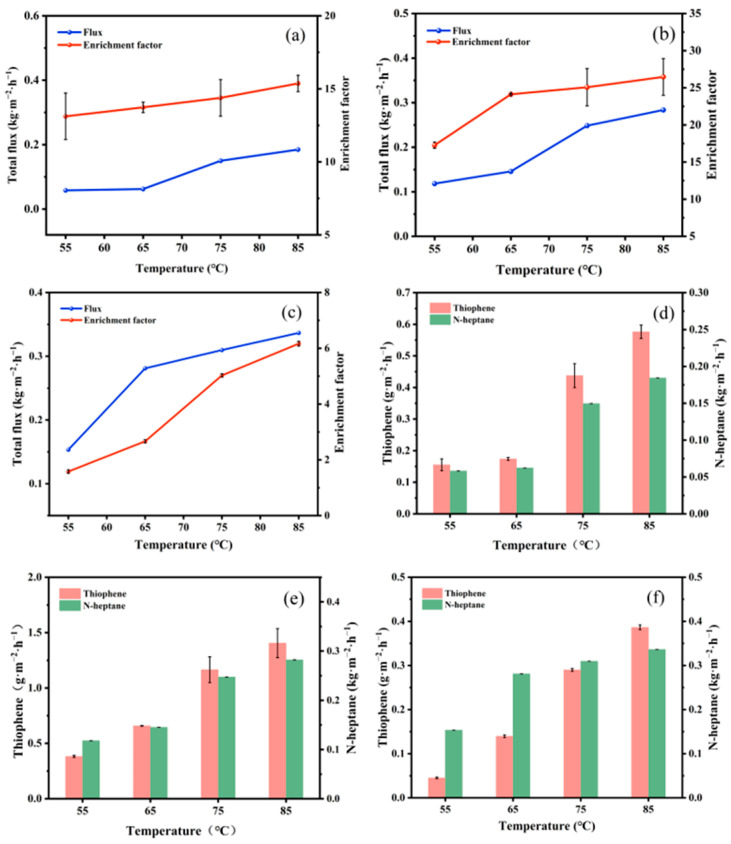
Influence of feed temperature on membrane performance. (**a**–**c**). Total flux and sulfur enrichment factor, (**d**–**f**) partial flux, (**a**,**d**) PTFEP/PVDF, (**b**,**e**) PTFBP/PVDF, and (**c**,**f**) POFPP/PVDF.

As illustrated in Figure 9a–c, the total flux and sulfur enrichment factor of PTFEP, PTFBP, and POFPP composite membranes exhibit an increasing tendency as the temperature rises. This can be attributed to the temperature elevation, which leads to a rise in the difference in partial pressure of components between the two sides of the membrane, thereby enhancing the driving force for transmembrane mass transfer, resulting in an augmented total flux [34]. The observed increase in the partial fluxes of thiophene and *n*-heptane in Figure 9d–f with temperature elevation further supports the aforementioned explanation.

**Figure 10:**
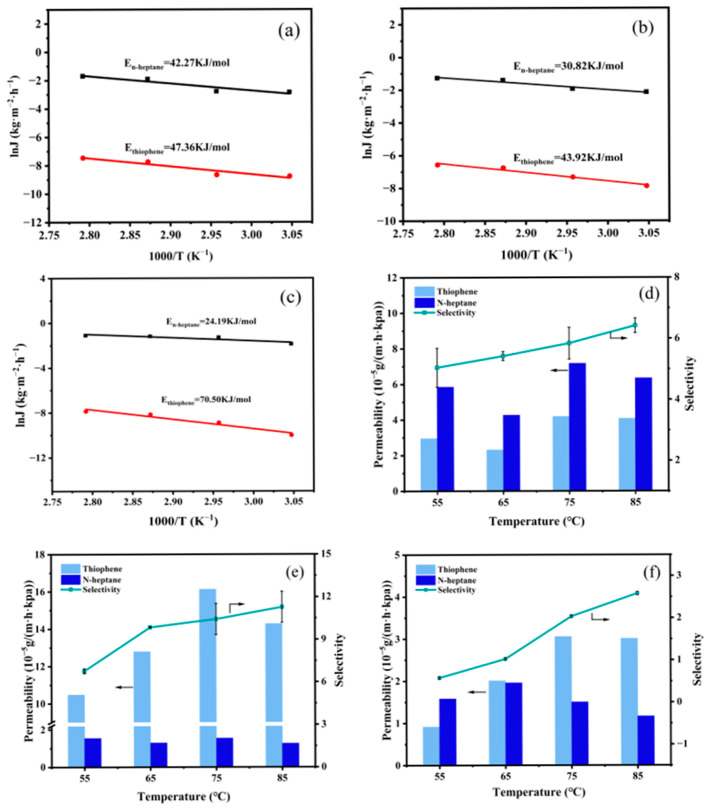
Influence of feed temperature on membrane performance. (**a**–**c**). Arrhenius curves, (**d**–**f**) permeability and selectivity, (**a**,**d**) PTFEP/PVDF, (**b**,**e**) PTFBP/PVDF, and (**c**,**f**) POFPP/PVDF.

The correlation between temperature and flux can be described using the Arrhenius formula, as demonstrated in Equation (2):

(2) $$J_{i}=J_{0i}EXP\left(\frac{-E_{p}}{RT}\right)$$

where J_i_ (kg·m^−2^·h^−1^) represents the partial flux of component i, *J_oi_* (kg·m^−2^·h^−1^) is the permeation flux constant, *E_p_* (kJ·mol^−1^) represents the apparent activation energy, *R* (J·mol^−1^·K^−1^) represents the gas molar constant, and *T* (°C) denotes the feed temperature. The calculated E_p_ of thiophene surpasses that of *n*-heptane, as depicted in Figure 10a–c. A higher *E_p_* indicates greater sensitivity to temperature variations and a more pronounced impact of temperature [30]. Consequently, as the temperature increases, the mass transfer rate of thiophene across the membrane surpasses that of *n*-heptane, resulting in an increase in the sulfur enrichment factor of the membrane. At this point, the pervaporation process is controlled by the dissolution process. The trends in membrane permeability and selectivity are illustrated in Figure 10d–f. With increasing temperature, the permeability of thiophene either remains stable or slightly increases, while the permeability of *n*-heptane decreases. The trend in selectivity aligns with the sulfur enrichment factor.

#### 3.2.2. Influence of Feed Concentration

**Figure 11:**
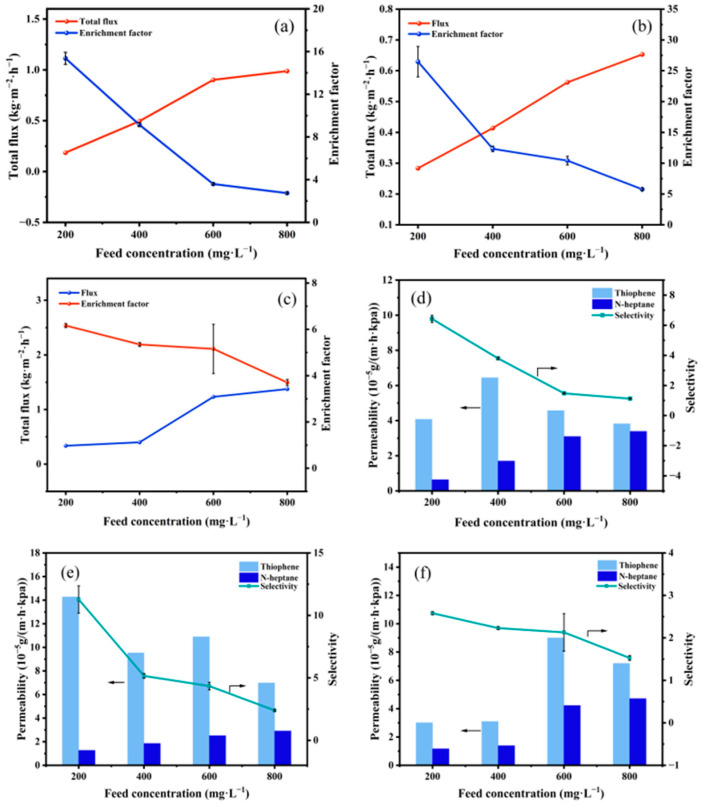
Influence of feed concentration on membrane performance. (**a**–**c**). Total flux and sulfur enrichment factor, (**d**–**f**) permeability and selectivity, (**a**,**d**) PTFEP/PVDF, (**b**,**e**) PTFBP/PVDF, and (**c**,**f**) POFPP/PVDF.

The pervaporation desulfurization performance of membranes at 85 °C with respect to feed concentration is elucidated in Figure 11. An increase in thiophene content in the feed solution results in a rise in total flux and a decrease in the sulfur enrichment factor for all membranes, as depicted in Figure 11a–c. The affinity of the membrane for thiophene leads to swelling and an expansion of the free volume within the membrane with increasing thiophene content in the feed solution [30]. Consequently, membrane flux increases. The increase in thiophene partial flux is relatively smaller due to the larger molecular dynamics diameter of thiophene compared to *n*-heptane, resulting in a decrease in the sulfur enrichment factor. As illustrated in Figure 11d–f, an increase in feed concentration leads to an essentially stable or increasing trend in thiophene and *n*-heptane permeability, while selectivity exhibits a decreasing trend, aligning with the sulfur enrichment factor trend.

#### 3.2.3. Influence of Side Group

**Figure 12:**
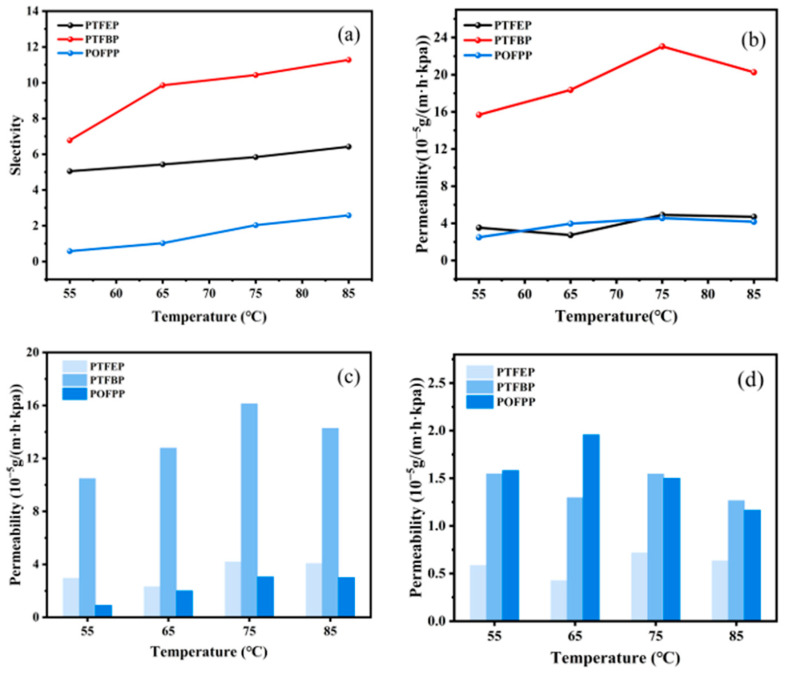
Influence of side group on membrane performance. (**a**) selectivity, (**b**) total permeability, (**c**) permeability of thiophene, and (**d**) permeability of n-heptane.

The separation performance of various membranes was compared at a constant thiophene feed concentration of 200 ppm, as shown in Figure 12. The results in Figure 12a,b indicate that the membranes exhibit the following trend in terms of selectivity and total permeability: POFPP < PTFEP < PTFBP. Figure 12c illustrates that the thiophene permeability follows the order: POFPP < PTFEP < PTFBP, aligning with the observed selectivity trend. Subsequently, in Figure 12d, the *n*-heptane permeability displays the trend PTFEP < PTFBP < POFPP. Notably, the *n*-heptane permeability of POFPP conforms to the selectivity trend, while that of PTFEP and PTFBP deviates from the expected trend, albeit marginally. All things considered, the total permeability of thiophene and *n*-heptane demonstrates general consistency with the selectivity trend.

The solubility parameters δ of thiophene with three types of POP were determined using the Fedors method [35]. The results show that the solubility parameters of thiophene, PTFEP, PTFBP, and POFPP are 19.89 MPa^0.5^, 16.43 MPa^0.5^, 16.75 MPa^0.5^, and 18.02 MPa^0.5^, respectively. According to the solubility parameter method for membrane material selection, a lower difference between the δ of the component to be separated and the membrane material indicates stronger solubility of the component on the membrane surface. Therefore, the separation efficiency of thiophene in different membranes should theoretically follow the order of PTFEP < PTFBP < POFPP, deviating slightly from the regular selectivity of different membranes. A higher SD leads to increased free volume inside the membrane; in addition, the reduction of T_g_ enhances the mobility of the POP molecular chain segments and decreases the diffusion resistance inside the membrane. By comparing the δ and T_g_ of different membranes, the diffusion rate inside the membrane can be ranked as POFPP < PTFEP < PTFBP, aligning with the regular permeability of different membranes. Compared to PTFEP, the T_g_ of PTFBP is notably lower, while the difference in solubility between the two is minimal. As a result, the intramembrane diffusion capability of PTFBP surpasses that of PTFEP. Furthermore, the wettability of PTFBP/PVDF for thiophene and *n*-heptane surpasses that of PTFEP/PVDF. Consequently, the dissolution process of thiophene and n-heptane occurs more effectively on the surface of PTFBP/PVDF membranes, enhancing the permeability of these compounds. Due to PTFBP’s solubility parameter being closer to that of thiophene and exhibiting a stronger affinity for thiophene, its selectivity towards model gasoline exceeds that of PTFEP. In comparison, POFPP demonstrated a higher T_g_ and lower SD, resulting in a weaker intra-membrane diffusion capacity. Although the solubility parameter of POFPP closely resembles that of thiophene, the wettability of POFPP/PVDF for thiophene and *n*-heptane is inferior to that of PTFBP/PVDF. Consequently, the surface solvation and adsorption processes of POFPP are weakened, leading to lower permeability and selectivity compared to PTFBP. The phenomenon is analyzed as follows: compared with PTFEP, PTFBP has a longer carbon chain on its side chain, leading to increased free volume and higher permeability. The fluorine atom within PTFBP is more electronegative [36], creating a shielding effect on the main-chain structure. However, the longer carbon chain on the side groups weakens this shielding effect, thereby enhancing the separation performance of PTFBP. On the other hand, POFPP has a significant number of fluorine atoms in its side chain, which are uniformly distributed. The low surface energy of C–F bonds enables the fluorine atoms in the side chains to aggregate towards the membrane surface. The high electronegativity of fluorine atoms creates a shielding effect on the main chain structure. This configuration makes it challenging for other atoms to be embedded, reducing the affinity with the components to be separated. Consequently, both the POFPP’s permeability and selectivity are lower.

#### 3.2.4. Comparison of Performance

In this paper, the comparison of the pervaporation desulfurization reported in the literature and our research is shown in Table 2. The best desulfurization performance of the PTFEP/PVDF composite membrane prepared in this work is close to that reported by Yang et al. [29]. Compared with other separation membranes, the desulfurization performance of the POFPP/PVDF composite membrane prepared in this work is poor, while the sulfur enrichment factor of the PTFBP/PVDF composite membrane has obvious advantages. This further confirms our origin, supposing that incorporating other fluorine-containing side groups into POP may obtain unforeseen outcomes.

## 4. Conclusions

A range of POP polymers with fluorine-containing side chains was synthesized successfully through molecular structure design. Subsequently, both homogeneous membranes and composite membranes were fabricated. The results show that the total flux and sulfur enrichment factor of the POP composite membrane increase as the temperature rises. Meanwhile, the total flux increases while the sulfur enrichment factor declines with increasing concentration. When the fluorine-containing side group of POP is the same, the extension of the carbon chain will enhance the permeability and selectivity of membranes. However, with increasing the fluorine content of the side group further, the permeability and selectivity of the POP membrane decrease. Therefore, the PTFBP/PVDF composite membrane shows the best desulfurization performance, which provides a new membrane material choice for the pervaporation desulfurization in the future.

## Figures and Tables

**Table 1:** Element content of POP composite membrane surface.

	F/%	C/%	O/%	N/%	P/%
PTFEP	29.23	20.98	10.77	5.07	5.01
PTFBP	27.02	36.11	13.38	5.04	5.30
POFPP	32.58	19.53	4.62	2.11	1.89

**Table 2:** Comparison of our prepared membranes and others.

Composite Membrane	Total Flux/(kg·m^−2^·h^−1^)	Enrichment Factor	Reference
PDMS/PSF	8.22	5.03	Liu [37]
PEG/PES	3.37	3.63	Kong [38]
MIL-101(Cr)PDMS	5.20	5.60	Yu [39]
CuBTC/PEBAX	16.45	4.04	Yu [40]
PEG@ZIF-8/PVDF	3.08	7.60	Sun [34]
PEBAX/PVDF	3.80	4.00	Liu [16]
CuBTC/PDMS	5.25	5.20	Yu [41]
PDMS-GNS/PVDF	6.22	3.58	Khodadadi [42]
Ag^+^@COFs/PEBAX	16.35	6.80	Pan [43]
PEBAX-Ag^+^@MOFs	22.11	5.92	Zhang [44]
PTFEP/PVDF	0.1	15.69	Yang [29]
PTFBP/PVDF	0.284	26.48	This work
PTFEP/PVDF	0.18	15.37	This work
POFPP/PVDF	0.34	6.17	This work

## Data Availability

The original contributions presented in this study are included in the article. Further inquiries can be directed to the corresponding authors.

## Abbreviations

POP	polyphosphazene
PTFEP	Poly[bis(trifluoroethoxy)phosphazene]
PTFBP	Poly[bis(trifluorobutoxy)phosphazene]
POFPP	Poly[bis(octafluoropentyloxy)phosphazene]
FCC	fluid catalytic cracking
PV	pervaporation
PI	polyimide
PEG	polyethylene glycol
PEBAX	poly(ether-block-amide)
PDMS	polydimethylsiloxane
EC	ethylcellulose
PDCP	Poly(dichlorophosphazene)
HCCP	Hexachlorocyclotriphosphazene
THF	tetrahydrofuran
SD	swelling degree
